# Detection of *Leishmania infantum* DNA and antibodies against *Anaplasma* spp., *Borrelia burgdorferi* s.l. and *Ehrlichia canis* in a dog kennel in South-Central Romania

**DOI:** 10.1186/s13028-020-00540-4

**Published:** 2020-08-03

**Authors:** Cristina Daniela Cazan, Angela Monica Ionică, Ioana Adriana Matei, Gianluca D’Amico, Clara Muñoz, Eduardo Berriatua, Mirabela Oana Dumitrache

**Affiliations:** 1 Department of Parasitology and Parasitic Diseases, University of Agricultural Sciences and Veterinary Medicine of Cluj-Napoca, Calea Mănăștur 3-5, 400372 Cluj-Napoca, Romania; 2 CDS-9, “Regele Mihai I al Romaniei” Life Science Institute, University of Agricultural Sciences and Veterinary Medicine of Cluj-Napoca, Calea Mănăștur 3-5, 400372 Cluj-Napoca, Romania; 3 Department of Microbiology, Immunology and Epidemiology, University of Agricultural Sciences and Veterinary Medicine of Cluj-Napoca, Calea Mănăștur 3-5, 400372 Cluj-Napoca, Romania; 4 Department of Animal Health, Faculty of Veterinary Science, Regional Campus of International Excellence ‘Campus Mare Nostrum’, University of Murcia, 30100 Murcia, Spain

**Keywords:** Canine vector-borne diseases, Dogs, Epidemiology, Kennel, *Leishmania infantum*

## Abstract

Canine vector-borne diseases are caused by pathogens transmitted by arthropods including ticks, mosquitoes and sand flies. Many canine vector-borne diseases are of zoonotic importance. This study aimed to assess the prevalence of vector-borne infections caused by *Dirofilaria immitis*, *Ehrlichia canis*, *Borrelia burgdorferi* sensu lato, *Anaplasma* spp. and *Leishmania infantum* in a dog kennel in Argeș County, Romania. Dog kennels are shelters for stray dogs with no officially registered owners that are gathered to be neutered and/or boarded for national/international adoptions by various public or private organizations. The international dog adoptions might represent a risk in the transmission of pathogens into new regions. In this context, a total number of 149 blood samples and 149 conjunctival swabs from asymptomatic kennel dogs were assessed using serology and quantitative real-time polymerase chain reaction. Antibodies against *B. burgdorferi* s.l. were detected in one dog (0.6%), anti-*Anaplasma* antibodies were found in five dogs (3.3%), while ten dogs (6.7%) tested positive for *D. immitis* antigen. Overall, 20.1% (30/149) of dogs were positive for *L. infantum* DNA. All samples were seronegative for anti-*Leishmania* antibodies. When adopting dogs from this region of Romania, owners should be aware of possible infection with especially *L. infantum*. The travel of infected dogs may introduce the infection to areas where leishmaniasis is not present.

## Findings

Canine vector-borne diseases (CVBDs) are currently an emerging problem due to the zoonotic character of some pathogens, for which dogs can serve as sentinels of human infection [1]. CVBDs are mainly caused by various species of bacteria and parasites, transmitted to dogs by arthropod vectors, especially ticks, mosquitoes or sand flies [2]. Among some of the major CVBD agents that can infect dogs are the nematode *Dirofilaria immitis*, bacteria such as *Ehrlichia canis*, *Borrelia burgdorferi* sensu lato, *Anaplasma phagocytophilum*, and the protozoan *Leishmania infantum* [3]. Evidence of northward and eastward expansion of *L. infantum* in non-endemic areas of Europe has been recorded, including in Romania [4]. In 2014, after 80 years with no data, a case of canine leishmaniasis (CanL) was described in Romania, raising the need for updates on the disease in the country [5]. In 2016, the first study to evaluate the prevalence of CanL in Romania by sensitive polymerase chain reaction (PCR) and serology revealed a 3.7% seropositivity and 8.7% PCR-positivity in the tested dogs (n = 80) [6]. In 2019, similar findings were reported. From two investigated dog kennels located in two different counties in South-Eastern Romania (Galaţi and Călăraşi), a CanL seroprevalence of 8.3% was present in Galaţi County (n = 60), while all samples from Călăraşi County (n = 50) were negative. The overall seroprevalence of the study was 4.54% (n = 110) [7]. Dog kennels are shelters for stray dogs with no officially registered owners that are gathered to be neutered and/or boarded for national/international adoptions by various public or private organizations. Co-infections with CVBD agents are common in kennel dogs, mostly because dogs are easily exposed to more than one vector species and the same vector species (particularly in case of ticks) may be infected with more than one pathogen [8]. Furthermore, apparently healthy dogs are of particular epidemiological importance, as they can act as reservoirs for human diseases [9].

The present study aimed to extend the current epidemiological knowledge on CVBDs in Romania in the context of national/international dog adoptions which might represent a risk in the transmission of pathogens into new regions.

**Fig. 1:**
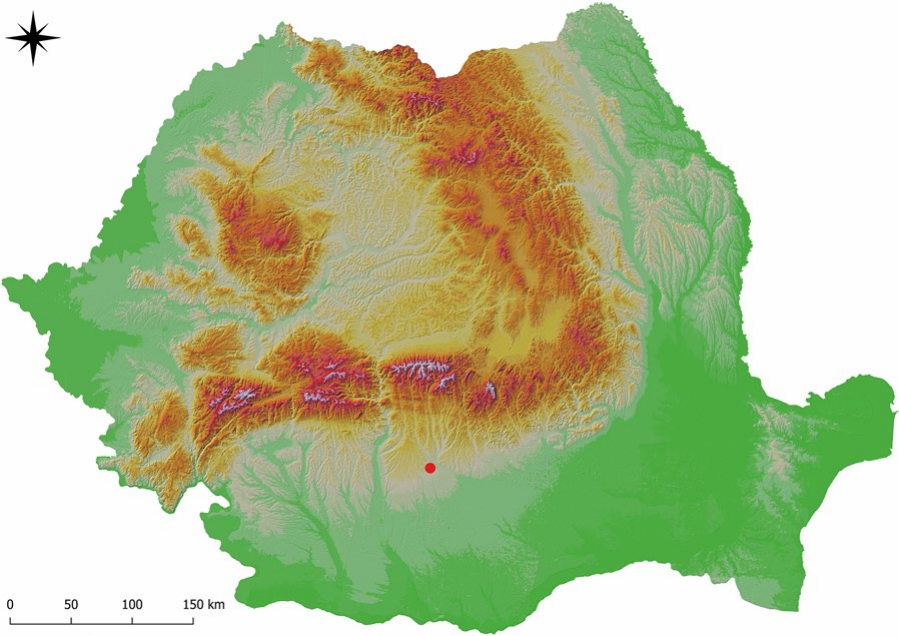
The sampling location in Argeş County, Romania (44.825 N, 24.800 E)

The study was performed during June–September 2017. Blood and conjunctival swab samples were collected from dogs (n = 149) located in a single kennel in Argeş County (44.825 N, 24.800 E) (Fig. 1), a geographical region that neighbors an area with recent local CanL reports [5, 6]. Prior to sampling, the dogs were examined for clinical signs of CanL, including lymphadenopathy, dermatitis, hair loss, cachexia and hepato-splenomegaly. The origin of the kennel dogs, prior of their gathering in the kennel, was known as local, free roaming dogs.

The occurrence of *Anaplasma* spp., *Borrelia burgdorferi* s.l., *E. canis* and *D. immitis* was assessed by using a serological rapid test, SNAP^®^ 4Dx^®^ (IDEXX Laboratories Inc., Westbrook, ME, USA) according to the manufacturer’s instructions.

Also, all serum samples were tested for the presence of anti-*L. infantum* antibodies by using a rapid test (SNAP^®^ Leishmania, IDEXX Laboratories Inc.) followed by the use of a commercial kit (INGEZIM LEISHMANIA 15.LSH.K1, Ingenasa, Spain) according to the manufacturer’s instructions.

Genomic DNA was isolated from both blood clots and swabs using a commercial kit (Isolate II Genomic DNA Kit, Bioline, London, UK) according to the manufacturer’s instructions. Prior to DNA isolation, the swabs were suspended in 300 µL 1× phosphate-buffered saline (PBS). All DNA samples were processed by quantitative real-time PCR (qPCR) amplification of the kinetoplast minicircle DNA of *L. infantum*, using the LEISH-1/LEISH-2 primer pair and TaqMan-MGB probe according to [10]. For the qPCR reaction, a positive control containing genomic target DNA and a negative control without DNA were included in order to assess the specificity of the reaction and the presence of cross-contamination.

Statistical analysis was performed using EpiInfo™ 7 software (https://www.cdc.gov/epiinfo/index.html, Centers for Disease Control and Prevention, USA). The frequency and prevalence of infection and their 95% confidence intervals were calculated. The differences among sex and age groups were assessed by Chi-square testing (α = 0.05) and correlations were evaluated by Spearman’s Rho.

No clinical signs of CanL or other diseases were observed.

The results of the SNAP^®^ 4Dx^®^ revealed antibodies against *B. burgdorferi* s.l. in one dog (0.6%; 95% CI 0.02–3.68%), anti-*Anaplasma* antibodies in five dogs (3.3%; 95% CI 1.10–7.66%), while ten dogs (6.7%; 95% CI 3.27–12.00%) tested positive for adult *D. immitis* female antigens. All samples (n = 149) tested negative for anti-*L. infantum* antibodies to both SNAP^®^*Leishmania* and INGEZIM Leishmania.

The qPCR screening revealed that 30 dogs (20.1%; 95% CI 14.02–27.48%) were positive for *L. infantum* DNA; 14 were positive on blood samples (9.4%; 95% CI 5.23–15.26%) and 17 were positive on conjunctival swab samples (11.4%; 95% CI 6.79–17.64%), with one animal expressing positive results for both the blood and swab sample.

The differences in prevalence among sex were not statistically significant (Table 1). Although a higher prevalence was noted in dogs older than 8 years of age as compared to younger dogs, the difference was not significant (Table 1).

**Table 1:** The statistical analysis of PCR-positive samples according to sex and age of the sampled dogs (n = 149)

	Frequency	Prevalence (%)	95% CI	P
Sex				
Male	18/89	20.2	12.45–30.07	1 (Χ^2^ = 1; d.f. = 1)
Female	12/60	20	10.78–32.33	
Age (years)				
≤ 3	9/47	19.1	9.15–33.26	0.061 (Χ^2^ = 5.586; d.f. = 2)
3–8	7/57	12.2	5.08–23.68	
≥ 8	14/45	31.1	18.17–46.65	

Among the *Leishmania*-positive dogs, three were also harboring a *D. immitis* infection. However, there was no significant correlation between the two pathogens (R = 0.066; P = 0.423).

Many studies on the prevalence of CVBDs worldwide have compared prevalence among asymptomatic and ill dogs, confirming the similar importance of both categories in the CVBDs transmission [9, 11–17]. Even though the importance of ill dogs in CVBDs transmission is more obvious, due to the presence of clinical signs, the asymptomatic dogs could be infected for months or even years, and still serve as reservoirs of pathogens to other hosts including humans [9].

In Romania, several studies targeting the occurrence of CVBPs in hosts and vectors have revealed a wide distribution, but with variable prevalence, according to various local ecological factors. In a molecular survey, *B. burgdorferi* s.l. was present in 1.4% of questing *Ixodes ricinus* ticks, with an average local prevalence ranging between 0.7 and 18.8% in all major regions of Romania [18]. In another study, *Borrelia* spp. DNA was identified in 138 of 534 (25.8%) questing *I. ricinus* ticks in eastern Romania [19]. The present study revealed antibodies against *B. burgdorferi* s.l. in only one dog (0.6%), but a low prevalence in canine hosts (6/1146; 0.5%) was also previously described in Romania [20].

Canine granulocytic anaplasmosis (CGA) is caused by *A. phagocytophilum*, the bacteria being transmitted by *I. ricinus* in Europe and infecting a wide range of domestic and wildlife hosts, including humans [21]. CGA has been reported in dogs from most regions of Romania, with an overall seroprevalence of 2.1% [20]. The overall prevalence of the infection in questing *I. ricinus* ticks was of 3.4%, with local prevalence values ranging between 0.2 and 22.4% [22].

In the present study no anti-*E. canis* antibodies were detected. Canine monocytic ehrlichiosis caused by *E. canis* is a disease transmitted by *Rhipicephalus sanguineus* s.l. ticks [20]. In Romania, an overall prevalence of *E. canis* of 2.1% (24/1146) was described in *R. sanguineus* s.l. ticks [20]. Seropositivity to *E. canis* is considered a risk factor for *D. immitis* and *L. infantum* infections [9].

The southern and southeastern areas of Romania are endemic for dirofilariasis caused by *D. immitis*, the dog heart worm [23]. Several studies have been conducted in order to evaluate the prevalence of heart worm infection. In a study evaluating 390 dogs from five regions of Romania, a 6.9% PCR positivity, and a 7.1% seropositivity were described [23].

Although other studies performed in Romania revealed seroprevalences against *L. infant*um, varying between 3.7 and 8.3% [6, 7], all samples in the present study were seronegative. However, *L. infantum* DNA was detected, in 20.1% (30/149) of the tested dogs and in 9.4% of the blood samples and 11.4% of the swab samples. Similar findings were described by Solano-Gallego et al. [11] when investigating dogs from Mallorca, Spain, where 37% of the sampled asymptomatic animals were PCR positive for the skin samples and/or conjunctival swabs, but seronegative. The PCR-positive and seronegative dogs are considered clinically healthy and should be retested in 6 to 12 months to assess the possible progression of the infection towards disease [24]. The *L. infantum* infection triggers a humoral response after the incubation time, which in general can vary between 3 weeks and 5 months. Thus, the detection of *L. infantum* DNA without seroconversion is a common finding [11]. Undoubtedly, the finding of antibodies against *L. infantum* indicates exposure to the parasite, but it is not clear if these dogs are immune or if they will develop the disease at some point. In the present study, the retesting of the seronegative and PCR-positive clinically healthy dogs was not possible, and further studies are needed in order to have a better understanding of this category of dogs.

The prevalence of CVBD infections in dog kennels is generally higher than the prevalence in the general dog population in a certain area. This is because stray dogs are much more exposed to pathogens before they are gathered and kept at a high population density in the kennels [25]. Therefore, the dog kennels may act as important sources of zoonotic diseases of veterinary and public health interest.

In the actual European context of international adoptions of kennel dogs, there is a permanent risk for spread of pathogens and zoonotic transmission. A detailed knowledge of the risk zones in Europe as a potential origin for stray dogs is important in the prevention of this potentially neglected category of source of infection represented by the apparently healthy kennel dogs.

The study revealed a high prevalence of *L. infantum* which appears to be widespread in Argeş County, Romania. Further studies are imperative to actively search for the sand fly vectors of CanL in the nearby areas, as well as to evaluate the potential neglected role of the asymptomatic dogs in the reemergence of CVBDs in Romania.

## Data Availability

All data generated or analysed during this study are included in this published article.

## Abbreviations

CVBDs: Canine vector-borne diseases
CanL: Canine leishmaniasis
*s.l.*: sensu lato
CGA: Canine granulocytic anaplasmosis
PBS: Phosphate-buffered saline
PCR: Polymerase chain reaction
